# Fibre-Cement Panel Ventilated Façade Smart Control System

**DOI:** 10.3390/ma14175076

**Published:** 2021-09-05

**Authors:** Anna Adamczak-Bugno, Grzegorz Świt, Aleksandra Krampikowska

**Affiliations:** Faculty of Civil Engineering and Architecture, Kielce University of Technology, 25-314 Kielce, Poland; gswit@tu.kielce.pl (G.Ś.); akramp@tu.kielce.pl (A.K.)

**Keywords:** ventilated façade, acoustic emission method, fibre-cement boards

## Abstract

This paper outlines a design for a fibre-cement panel ventilated façade smart control system based on the acoustic emission method. The paper also provides methodology and test results, as well as statistical analysis of the three-point bending results with AE signal acquisition as a basis for the development of the system in question. The test items were samples cut from a full-size fibre-cement panel for interior and exterior use, according to the standard guidelines. The recorded acoustic emission signals were classified statistically into four classes, which were assigned to the processes occurring in the material structure as a result of the applied load. The system development was based on the differences between the characteristics of the individual signal classes and their number for each test case, as well as on the different distribution of successive classes over time. Given the results of the tests and the resulting conclusions indicating the applicability of the acoustic emission method (based on signal classification using the k-means algorithm for the assessment of variations in the mechanical parameters of cement-fibre composites), a methodology for such assessment was therefore developed. The approach proposed is a reasonable method for assessing the variation in mechanical parameters of fibre-cement panels on the basis of the parameters determined by the non-destructive method indicated.

## 1. Introduction

Fibre-reinforced cement-based products have been used in the construction industry for over a century. The inventor of the first fibre-cement panels was an engineer from the Czech Republic, Ludwik Hatschek [1,2,3,4], who led the development and patenting of the technology to produce this type of composite. The original product, called ‘Eternit’, demonstrated high strength and durability while being light, moisture-resistant, and non-combustible. The combination of these parameters in fibre-cement panels was an outstanding innovation globally. The fibre-cement panels have proven to be the most popular roofing material in the world over the last century [5]. The backlash came when one component, asbestos, was found to be carcinogenic [6]. In the 1990s, work began on replacing the toxic component. Since then, the cement matrix has been reinforced with fibres that do not pose a health risk to users, mainly cellulose fibres. Fibre-cement panels available on the market today may include cement, cellulose fibres, synthetic fibres, and various additives and admixtures [7] which makes them a different type of building product in relation to the original. Limestone dust, mica, perlite, kaolin, and microspheres are the most common additives and fillers in fibre-cement panels [8,9,10,11,12]. Products containing recycled materials (cellulose recycling) are also available [13]. All types of fillers used contribute to the innovative properties of fibre-cement products and are part of sustainable development.

The cement-fibre panels are a material exposed to defects and damage, both during the manufacturing process and in service. This fact significantly affects their properties and durability parameters. The production of panels is a complex process governed by the properties and quantities of the individual components and how they are incorporated into the finished product [14,15,16,17,18]. During the consecutive production stages, the panels are exposed to defects and damage in the form of large pores, delamination, cracks and discontinuities in the reinforcement fibre distribution. In service conditions, the fibre-cement panels are exposed to variable atmospheric and environmental conditions, both in terms of temperature and humidity, in the form of significant temperature fluctuations, including transitions through 0 °C in a daily cycle (cyclic freeze-thaw phenomenon), the likelihood of chemical aggression in the form of acid rainfall, and exposure to ultraviolet rays [19,20,21,22,23,24,25]. In addition, the fibre-cement cladding panels are exposed to extreme service conditions. The most destructive of these is heat and direct fire exposure in a fire scenario. Service impacts in the form of horizontal force are also potentially damaging from a strength perspective. The occurrence of the above-mentioned conditions during the service life of the panels is related to the fact that they are used as external façade cladding in the ventilated façade system, as balcony and terrace cover panels, and as wall finishes inside buildings [26].

When these factors occur, there is a high probability that the cement-fibre panel structure will change, resulting in a deterioration of the performance and durability properties [27,28,29,30]. Therefore, cladding panels should meet the requirements set out in the standards and legislation [31], taking into account the impacts indicated. Determining the extent to which fibre-cement panels are affected by service conditions is important, particularly for the external parts of buildings, as this has a direct impact on the safe use of a building. This issue seems to be particularly relevant for tall and high-rise buildings. The question of providing a proven, reliable, and durable cladding material is therefore indisputable. The mere determination of the impact of the presented conditions on the change of strength parameters of the cement-fibre panels is one of the basic conditions of safe operation. However, it is important to stress that a very significant contribution to the final product quality and durability is also attributable to testing at the product production stage [32,33,34].

Fibre-cement is subject to the same processes as other cement-based products. One of the most intensive is carbonatization, the intensity of which depends, among other things, on the concentration of carbon dioxide. The way the material behaves during use is also affected by its layered structure. The studies on cement-fibre materials show that in the first years of service, the reaction between the calcium phases and carbon dioxide from air and water has a positive effect on the bonding between the product layers. However, after only about five years, reactions of the carbon with hydration products result in moisture migration tracks in the material. Long-term exposure of the panels to weather ultimately leads to a reduction in the bonding between the layers and the formation of a discontinuity in the matrix. Cellulose fibres also become particularly vulnerable due to increased moisture movement in the material and freeze-thaw cycles. The gradual depolymerisation of the fibres, resulting in, among other things, a decrease in mechanical strength, is a direct effect of these interactions. It is estimated that after about 15 years of service, the strength of cement-fibre panels decreases by up to 40% [31,32].

Due to the processes taking place in the material of cement-cellulose boards and due to the operational conditions to which they are exposed, it is necessary to at least periodically monitor the buildings made with their use. This type of control should make it possible to detect a possible decrease in the mechanical parameters of the boards.

Taking into account the conclusions of the literature review, steps were taken to select a non-destructive method that would allow the control of the built-in boards. The result of the carried-out work is the system for assessing the change in mechanical parameters of fiber-cement panels, presented in the article, based on the acoustic emission method. The paper also presents the research that is the basis for the development of the final diagnostic methodology.

## 2. Materials and Methods

The tests prior to the system development to assess the change in mechanical performance of the fibre-cement panels involved three-point bending tests on samples cut from full-size panels together with acoustic emission (AE) signal acquisition. The bending configuration and dimensions of the test pieces were chosen based on the standard guidelines.

The test pieces were subjected to environmental conditions (soaking in water for 1 h and 24 h, cyclic freeze-thaw in an air-water environment for 10 cycles, 25 cycles, 50 cycles, and 100 cycles) and extreme conditions (burning in a laboratory oven for 3 h at 230 °C, exposure to direct fire for 2.5 min, 5.0 min, 7.5 min, 10.0 min), and were also tested in air-dry condition. The acoustic emission method in the energy approach was used as the main testing method to determine the change in mechanical parameters of cement-fibre composites.

**Figure 1:**
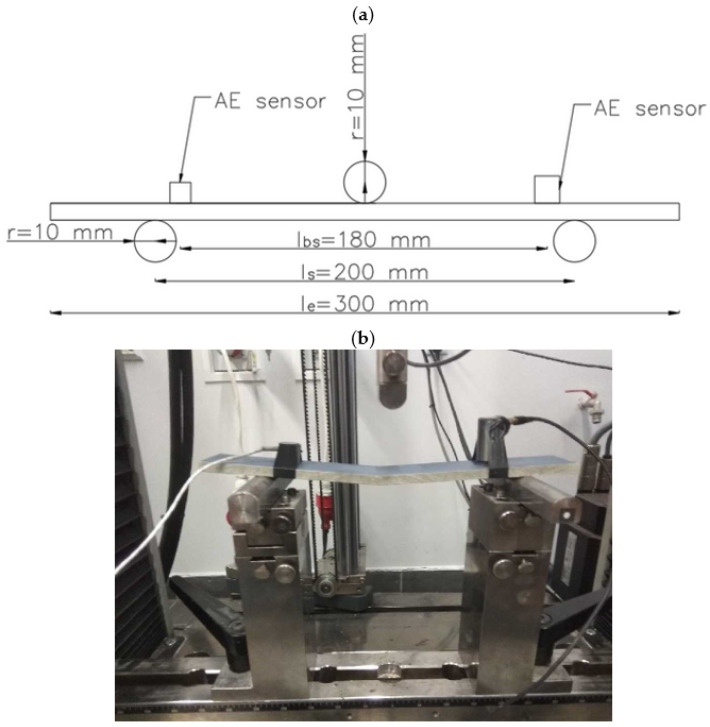
Test stand diagram: (**a**) load diagram; (**b**) a photograph of one of the samples.

The scheme of the test stand and a photograph of one of the tested samples are shown in Figure 1. Cuboidal 300 × 50 × 8 mm samples were cut from 1.25 × 3.10 m and 8 mm thick panels for three-point bending. Load *F*, displacement and acoustic emission signals AE were recorded during the tests. The distance between the support axes (l_s_) was 200 mm. A Zwick Roell testing machine was used in the tests. The constant loading speed was 0.1 mm/s.

According to the manufacturer’s declaration, the boards from which the test elements were cut out consisted of Portland cement, mineral binders, cellulose fibers, color dyes, and admixtures. Accurate quantitative and qualitative data on the composition are protected by patent law. The declared technical parameters of the board are presented in Table 1.

Fourteen standard AE parameters were used to determine the change in mechanical parameters:

1. Duration;
2. Rise time;
3. Decay time;
4. RMS;
5. Counts;
6. Counts to peak;
7. Amplitude;
8. Energy;
9. Average frequency;
10. Reverberation frequency;
11. Initiation frequency;
12. Absolute energy;
13. Signal strength;
14. ASL.

These parameters were used to classify the signals using pattern recognition methods with arbitrary class division (*USPR*). Scattergram of signal strength and duration were used to present the results of the tests after analysis with the created reference signal database. The advantage of creating a database of reference signals is the fact of assigning invariable characteristics to individual classes, marked by graphic symbols and colour. As a result, we can use selected parameters (preferably depicting the transformation of destructive processes) rather than all AE parameters to analyse the processes taking place, which facilitates the interpretation of the results by people not trained in acoustic emission testing. According to Świt et al. [33], analysis using the parameters of signal power and duration illustrates well the distribution of signals of the different classes and makes it possible to observe the differences between them.

To assess the change in mechanical parameters as affected by service (environmental and extreme) conditions, a three-point bending test was conducted during the *MOR* flexural strength test. The standard parameter for assessing the change (deterioration) of the mechanical parameters of the tested cement-fibre panels are the classes of acoustic emission signals with the destructive processes assigned to them, namely fibre cracking and fibre pull-out from the cement matrix. The decline in recorded events of this nature allowed assessment of the extent to which mechanical parameters change when exposed to service conditions. The other classes serve as a preliminary confirmation of the presence of other destructive processes in the tested pieces.

On the basis of the preliminary tests performed, it was confirmed that the external panels have the mechanical parameters as declared by the manufacturers. It is important to note, however, that the fibre-cement panel compositions and their technological process are strictly protected by the manufacturers, and information on specific components, their quantities and suppliers, and production details are restricted extensively. The fibre-cement panels tested were made using basic components, such as Portland cement CEM I 42.5N, cellulose fibres, synthetic fibres PVA and additives, such as limestone dust. The coloured panels have additional colouring admixtures. All panels were manufactured according to the Hatschek process.

The test samples were cut in one direction, parallel to the length of the panel, avoiding the 50 mm edge zone of the panel.

The changes in the mechanical parameters of the fibre-cement panels under environmental and extreme service conditions were assessed for the following test cases:

- Air-dry condition (no destruction, reference condition, referential);
- Soaking in water for 1 h;
- Soaking in water for 24 h;
- 25 soaking-drying cycles;
- 50 bath-drying cycles;
- 10 freeze-thaw cycles;
- 25 freeze-thaw cycles;
- 50 freeze-thaw cycles;
- 100 freeze-thaw cycles;
- Direct exposure to flame (resulting in a temperature of up to 400 °C) for 2.5 min;
- Direct exposure to flame (resulting in a temperature of up to 400 °C) for 5 min;
- Direct exposure to flame (resulting in a temperature of up to 400 °C) for 7.5 min;
- Direct exposure to flame (resulting in a temperature of up to 400 °C) for 10 min;
- Temperature of 230 °C for 3 h.

The creation of a reference signal database in the acoustic emission method involves several steps. These include:

- Signal generation under laboratory conditions during the destruction of standard samples in a specified manner;
- Comparison of signals from the destruction of model samples subjected to different destruction processes;
- Verification of the reference signals generated by various types and lengths of composite panel loaded to destruction;
- Final verification on components during standard operation (this step will be performed during further tests).

The next step was to adopt the standard parameters needed to create a reference signal database, namely:

- The assumed number of destructive processes (based on literature and own research);
- Measures of distance between clusters–in this case Euclidean space with time distribution;
- The number of iterations necessary to determine the optimal number of classes (min. 1,000,000).

The reference file with four classes obtained in this way was then verified on the samples in laboratory tests with single destructive processes prevailing.

As a result of the work performed, a database of reference signals was obtained for assessing the technical condition of the panels. Using the reference signal database, the individual AE parameters are only an illustration of the processes taking place and not a source for analysis. Therefore, it is important to apply *BIG DATA* analyses to create *“BLACK BOX”*, which can be used for structural health analysis by people without academic knowledge of AE.

The number of classes was imposed by a statistical software using the k-means algorithm for the analysis of AE signals based on the literature reports. Another criterion to explain the imposed number of classes was how well the individual signals matched the correct classes, which in this case was over 90%.

## 3. Results

After dividing the recorded AE signals into four classes, using the *k-means* algorithm, two of the fourteen acoustic emission parameters–the signals strength and their duration –were used as illustrations of the processes taking place. Individual AE signal classes assigned, based on preliminary tests, to processes occurring in the structure of the tested material include:

- Class 1 (green colour)–micro-crack initiation;
- Class 2 (red colour)–crack development and spread;
- Class 3 (blue)–material delamination, fibre separation;
- Class 4 (yellow colour)–fibre breakage, material deterioration;

The paper presents graphs of the descriptors over time, including their division into classes, for mock samples from each of the tested series. The results obtained for samples within the group were similar.

### 3.1. AE Signal Grouping Results

#### 3.1.1. Signal Analysis for Research Case A_1_

**Figure 2:**
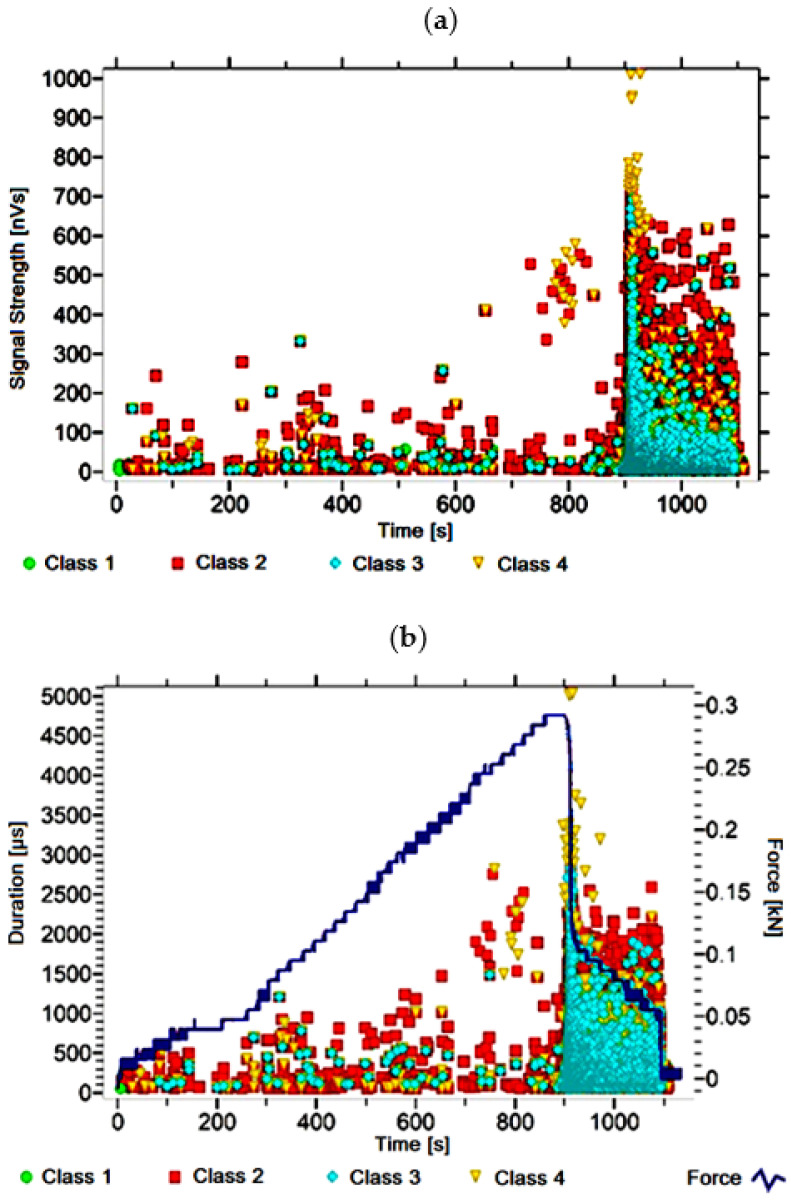
Graphs of acoustic emission signals for an exemplary sample of A_1_ series: (**a**) signal strength over time; (**b**) distribution of the duration of the signals over time with the force increment curve.

For the sample from the A_1_ (Figure 2) test case, signals of all four classes were observed from the start of each recorded waveform. The signals corresponding to the initiation of microcracks appear from the start of the test until they reach 600 s; their power is up to 50 nVs and their duration up to 120 µs. The signals linked to crack development and spread are recorded throughout the measurement and, in most cases, are characterised by a power of up to 650 nVs and a duration of up to 2700 µs. Events corresponding to material delamination and fibre detachment were also recorded from the start of the test, but their intensity increases after 900 s. The strength of the signals associated with the indicated processes reaches 700 nVs. In turn, the duration of events of this class reaches 3000 µs. The power of the signals associated with fibre breakage and component destruction is up to 1000 nVs and their duration is generally up to 4500 µs.

#### 3.1.2. Signal Analysis for Research Case A_2_

**Figure 3:**
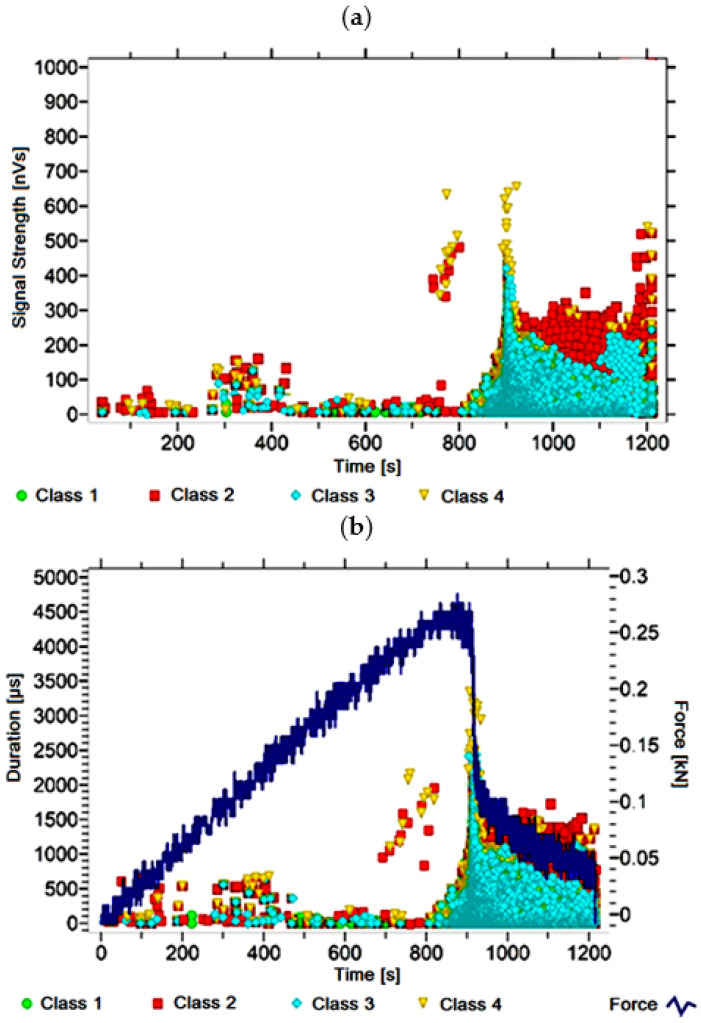
Graphs of acoustic emission signals for an exemplary sample of A_2_ series: (**a**) signal strength over time; (**b**) distribution of the duration of the signals over time with the force increment curve.

For the sample from the A_2_ (Figure 3) test case, signals of all four classes were observed from the start of each recorded waveform. The signals corresponding to the initiation of microcracks appear from the start of the test until they reach 650 s; their power is up to 130 nVs and their duration up to 200 µs. The signals linked to crack development and spread are recorded throughout the measurement and, in most cases, are characterised by a power of up to 550 nVs and a duration of up to 1700 µs. Events corresponding to material delamination and fibre detachment were also recorded from the start of the test, but their intensity increases after 800 s. The strength of the signals associated with the indicated processes reaches 500 nVs. In turn, the duration of events of this class reaches 2500 µs. The power of the signals associated with fibre breakage and component destruction is up to 700 nVs and their duration is 4000 µs.

#### 3.1.3. Signal Analysis for Research Case A_3_

**Figure 4:**
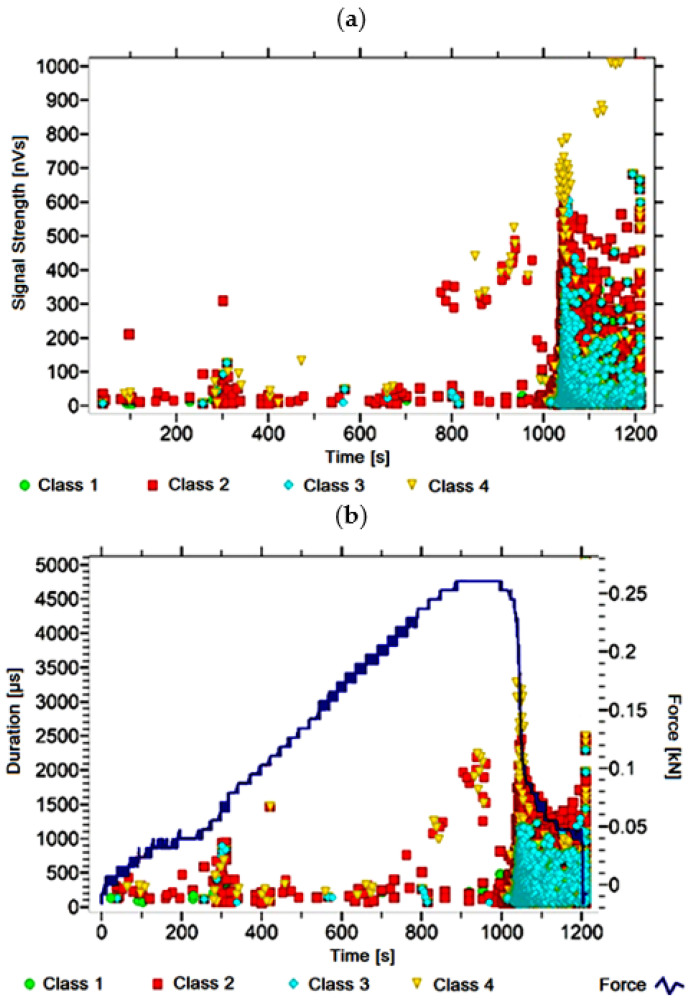
Graphs of acoustic emission signals for an exemplary sample of A_3_ series: (**a**) signal strength over time; (**b**) distribution of the duration of the signals over time with the force increment curve.

For the sample from the A_3_ (Figure 4) test case, signals of all four classes were observed from the start of each recorded waveform. The signals corresponding to the initiation of microcracks appear from the start of the test until they reach 1000 s; their power is up to 45 nVs and their duration up to 150 µs. The signals linked to crack development and spread are recorded throughout the measurement and, in most cases, are characterised by a power of up to 600 nVs and a duration of up to 2500 µs. Events corresponding to material delamination and fibre detachment were also recorded from the start of the test, but their intensity increases after 1000 s. The strength of the signals associated with the indicated processes reaches 700 nVs. In turn, the duration of events of this class reaches 2500 µs. The power of the signals associated with fibre breakage and component destruction is even up to 1000 nVs and their duration is 3500 µs.

#### 3.1.4. Signal Analysis for Research Case A_4_

**Figure 5:**
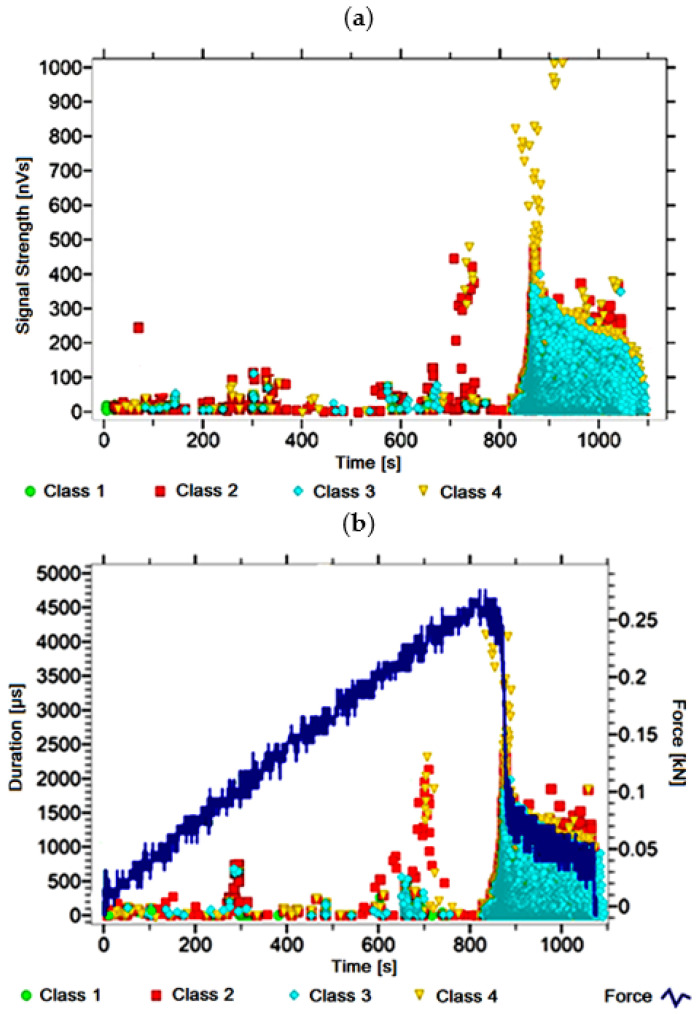
Graphs of acoustic emission signals for an exemplary sample of A_4_ series: (**a**) signal strength over time; (**b**) distribution of the duration of the signals over time with the force increment curve.

For the sample from the A_4_ (Figure 5) test case, signals of all four classes were observed from the start of each recorded waveform. The signals corresponding to the initiation of microcracks appear from the start of the test until they reach 600 s; their power is up to 60 nVs and their duration up to 200 µs. The signals linked to crack development and spread are recorded throughout the measurement and, in most cases, are characterised by a power of up to 500 nVs and a duration of up to 2100 µs. Events corresponding to material delamination and fibre detachment were also recorded from the start of the test, but their intensity increases after 800 s. The strength of the signals associated with the indicated processes reaches 450 nVs. In turn, the duration of events of this class reaches 2000 µs. The power of the signals associated with fibre breakage and component destruction is even up to 1000 nVs and their duration is 4500 µs.

#### 3.1.5. Signal Analysis for Research Case A_5_

**Figure 6:**
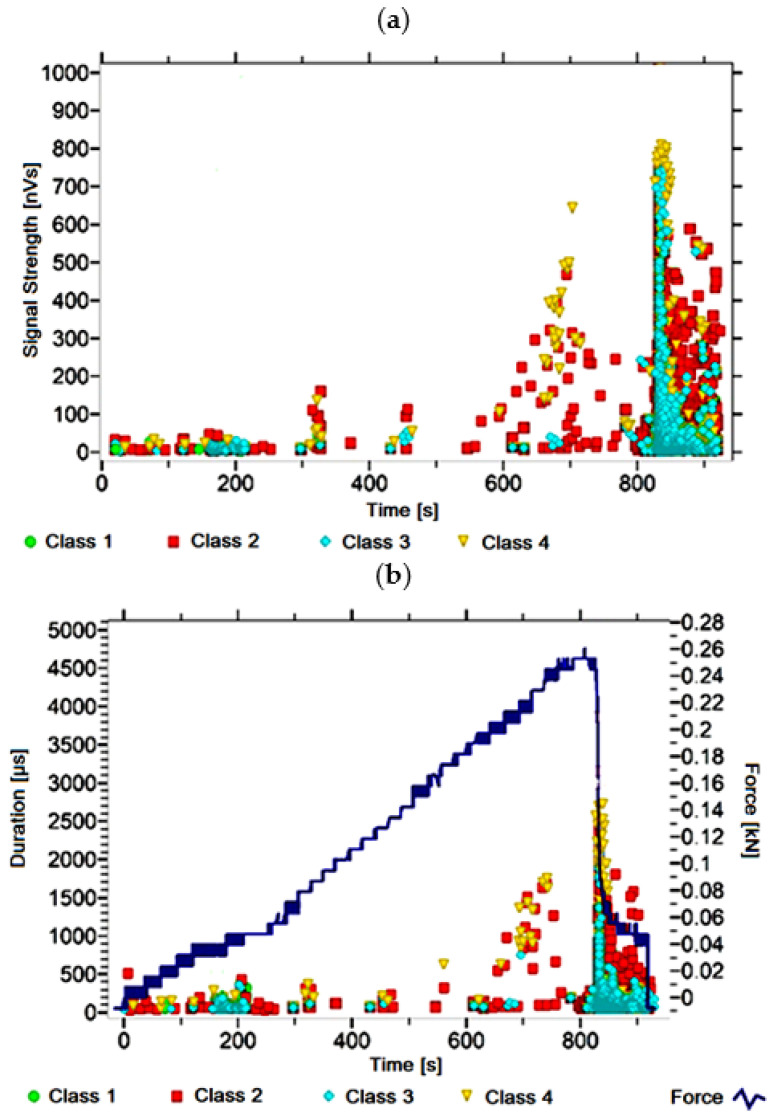
Graphs of acoustic emission signals for an exemplary sample of A_5_ series: (**a**) signal strength over time; (**b**) distribution of the duration of the signals over time with the force increment curve.

For the sample from the A_5_ (Figure 6) test case, signals of all four classes were observed from the start of each recorded waveform. The signals corresponding to the initiation of microcracks appear from the start of the test until they reach 400 s; their power is up to 50 nVs and their duration up to 150 µs. The signals linked to crack development and spread are recorded throughout the measurement and are characterised by a power of up to 600 nVs and a duration of up to 2000 µs. Events corresponding to material delamination and fibre detachment were also recorded from the start of the test, but their intensity increases after 800 s. The strength of the signals associated with the indicated processes reaches 800 nVs. In turn, the duration of events of this class reaches 2300 µs. The power of the signals associated with fibre breakage and component destruction is even up to 1000 nVs and their duration is 3000 µs.

#### 3.1.6. Signal Analysis for Research Case A_6_

**Figure 7:**
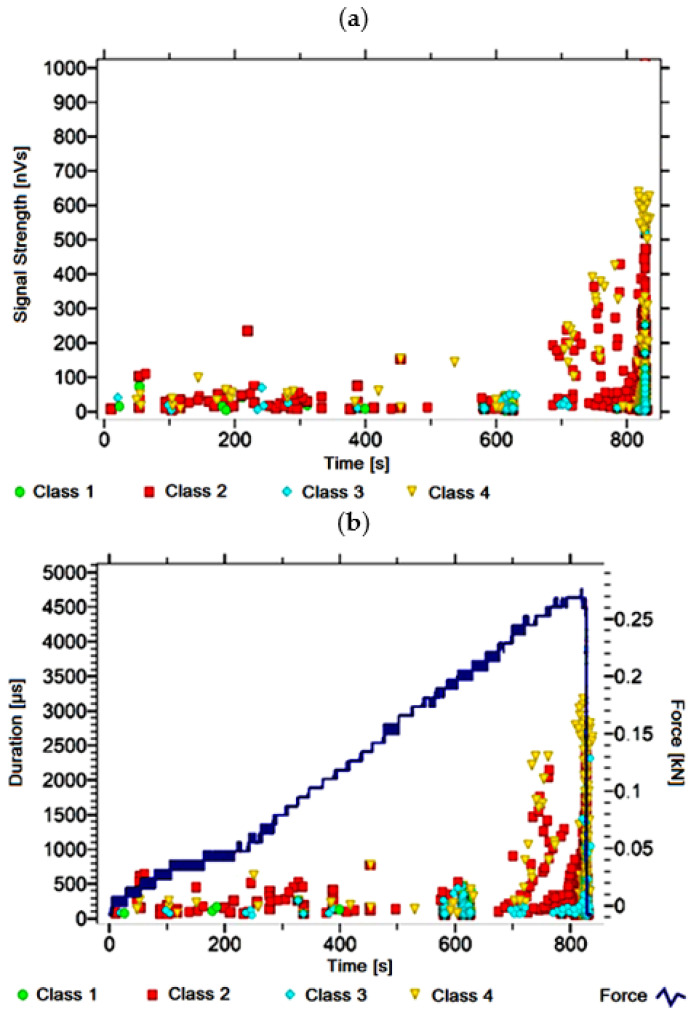
Graphs of acoustic emission signals for an exemplary sample of A_6_ series: (**a**) signal strength over time; (**b**) distribution of the duration of the signals over time with the force increment curve.

For the sample from the A_6_ (Figure 7) test case, signals of all four classes were observed from the start of each recorded waveform. The signals corresponding to the initiation of microcracks appear from the start of the test until they reach 400 s; their power is up to 50 nVs and their duration up to 200 µs. The signals linked to crack development and spread are recorded throughout the measurement and, in most cases, are characterised by a power of up to 500 nVs and a duration of up to 2200 µs. Events corresponding to material delamination and fibre detachment were also recorded from the start of the test, but their intensity increases after 700 s. The strength of the signals associated with the indicated processes reaches 400 nVs. In turn, the duration of events of this class reaches 2500 µs. The power of the signals associated with fibre breakage and component destruction exceeds 600 nVs and their duration is 3000 µs.

#### 3.1.7. Signal Analysis for Research Case A_7_

**Figure 8:**
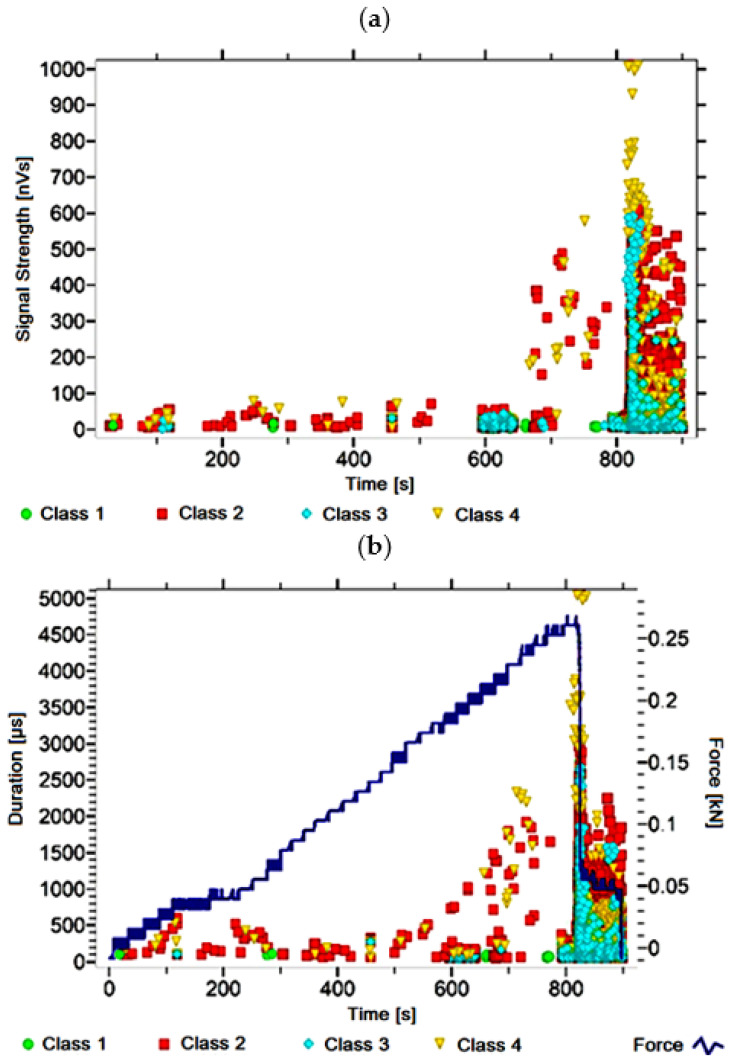
Graphs of acoustic emission signals for an exemplary sample of A_7_ series: (**a**) signal strength over time; (**b**) distribution of the duration of the signals over time with the force increment curve.

For the sample from the A_7_ (Figure 8) test case, signals of all four classes were observed from the start of each recorded waveform. The signals corresponding to the initiation of microcracks appear from the start of the test until they reach 700 s; their power is up to 45 nVs and their duration up to 170 µs. The signals linked to crack development and spread are recorded throughout the measurement and are characterised by a power of up to 600 nVs and a duration of up to 2300 µs. Events corresponding to material delamination and fibre detachment were also recorded from the start of the test, but their intensity increases at around 800 s. The strength of the signals associated with the indicated processes reaches 600 nVs. In turn, the duration of events of this class reaches 3000 µs. The power of the signals associated with fibre breakage and component destruction is even up to 1000 nVs and their duration is 5000 µs.

#### 3.1.8. Signal Analysis for Research Case A_8_

**Figure 9:**
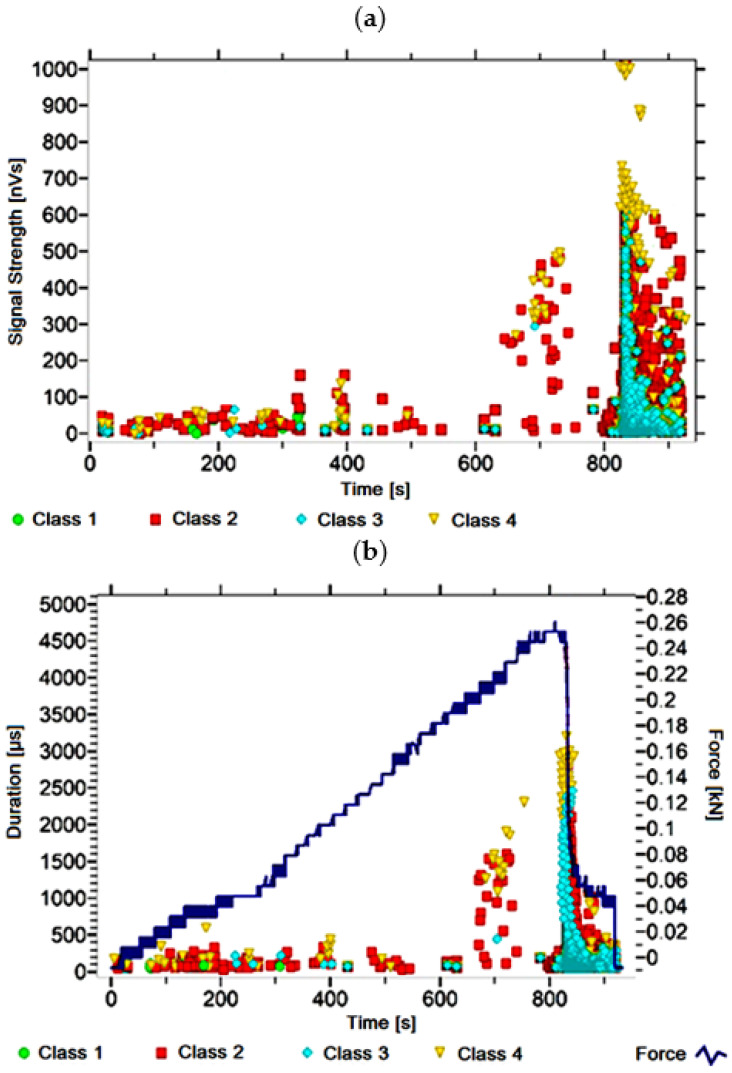
Graphs of acoustic emission signals for an exemplary sample of A_8_ series: (**a**) signal strength over time; (**b**) distribution of the duration of the signals over time with the force increment curve.

For the sample from the A_8_ (Figure 9) test case, signals of all four classes were observed from the start of each recorded waveform. The signals corresponding to the initiation of microcracks appear from the start of the test until they reach 400 s; their power is up to 50 nVs and their duration up to 130 µs. The signals linked to crack development and spread are recorded throughout the measurement and are characterised by a power of up to 600 nVs and a duration of up to 2000 µs. Events corresponding to material delamination and fibre detachment were also recorded from the start of the test, but their intensity increases after 800 s. The strength of the signals associated with the indicated processes reaches 600 nVs. In turn, the duration of events of this class reaches 2500 µs. The power of the signals associated with fibre breakage and component destruction is even up to 1000 nVs and their duration is 3000 µs.

#### 3.1.9. Signal Analysis for Research Case A_9_

**Figure 10:**
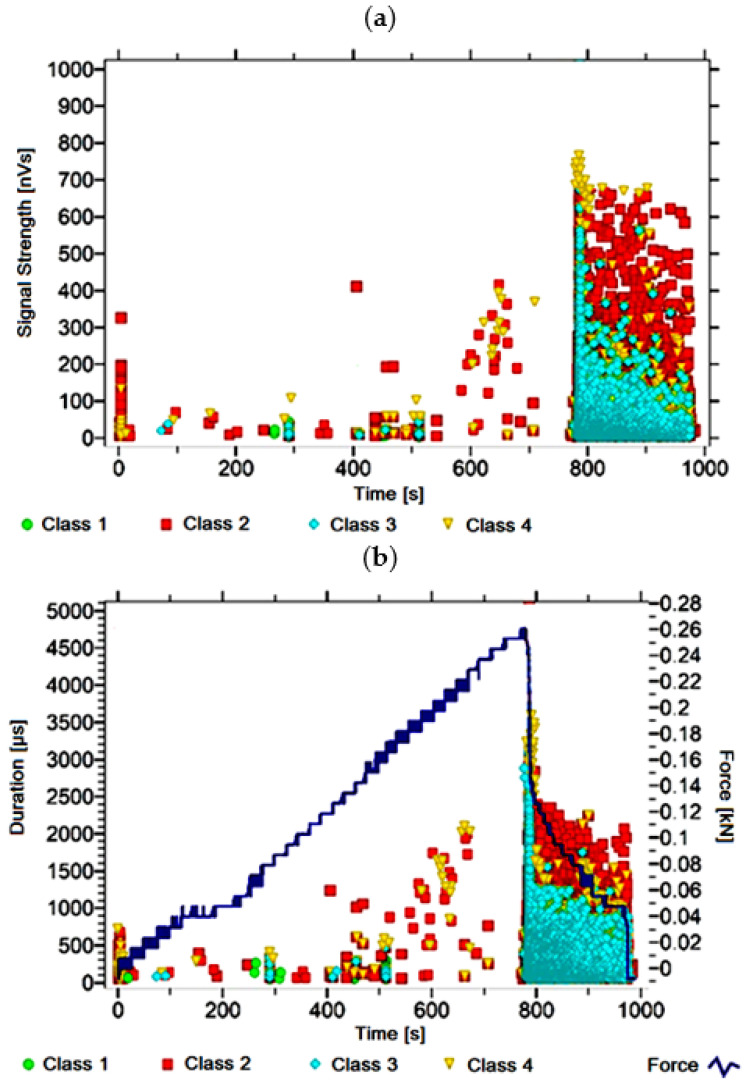
Graphs of acoustic emission signals for an exemplary sample of A_9_ series: (**a**) signal strength over time; (**b**) distribution of the duration of the signals over time with the force increment curve.

For the sample from the A_9_ (Figure 10) test case, signals of all four classes were observed from the start of each recorded waveform. The signals corresponding to the initiation of microcracks appear from the start of the test until they reach 500 s; their power is up to 60 nVs and their duration up to 250 µs. The signals linked to crack development and spread are recorded throughout the measurement and are characterised by a power of up to 700 nVs and a duration of up to 2500 µs. Events corresponding to material delamination and fibre detachment were also recorded from the start of the test, but their intensity increases at around 800 s. The strength of the signals associated with the indicated processes reaches 700 nVs. In turn, the duration of events of this class reaches 3000 µs. The power of the signals associated with fibre breakage and component destruction reaches up to 800 nVs and their duration is 4000 µs.

#### 3.1.10. Signal Analysis for Research Case A_10_

**Figure 11:**
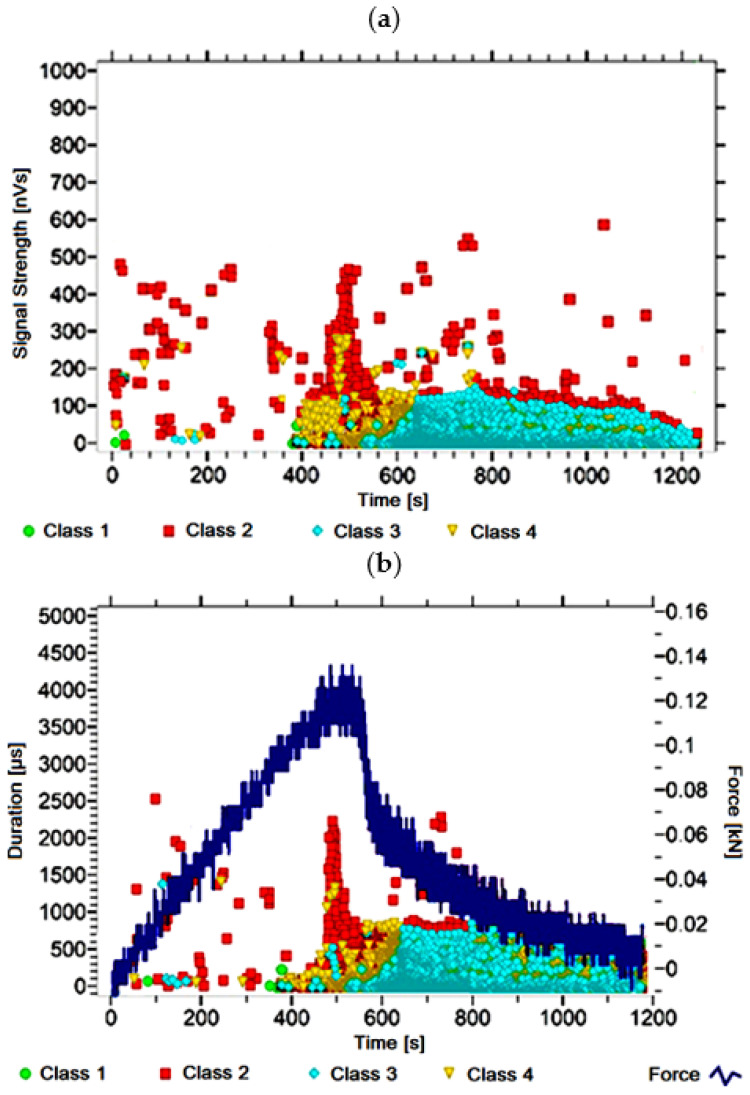
Graphs of acoustic emission signals for an exemplary sample of A_10_ series: (**a**) signal strength over time; (**b**) distribution of the duration of the signals over time with the force increment curve.

For the sample from the A_10_ (Figure 11) test case, signals of 1 and 2 classes were observed from the start of each recorded waveform. Class 3 and 4 signals are intensified at around 400 s. Signals corresponding to the initiation of microcracks occur up to about 400 s; their power is up to 50 nVs and their duration up to 250 µs. The signals linked to crack development and spread are recorded throughout the measurement and are characterised by a power of up to 500 nVs and a duration of up to 2500 µs. The intensity of events corresponding to material delamination and fibre detachment increases after 400 s. The strength of the signals associated with the indicated processes reaches 200 nVs. In turn, the duration of events of this class is 1300 µs. The power of the signals associated with fibre breakage and component destruction is up to 300 nVs and their duration is up 1500 µs.

#### 3.1.11. Signal Analysis for Research Case A_11_

**Figure 12:**
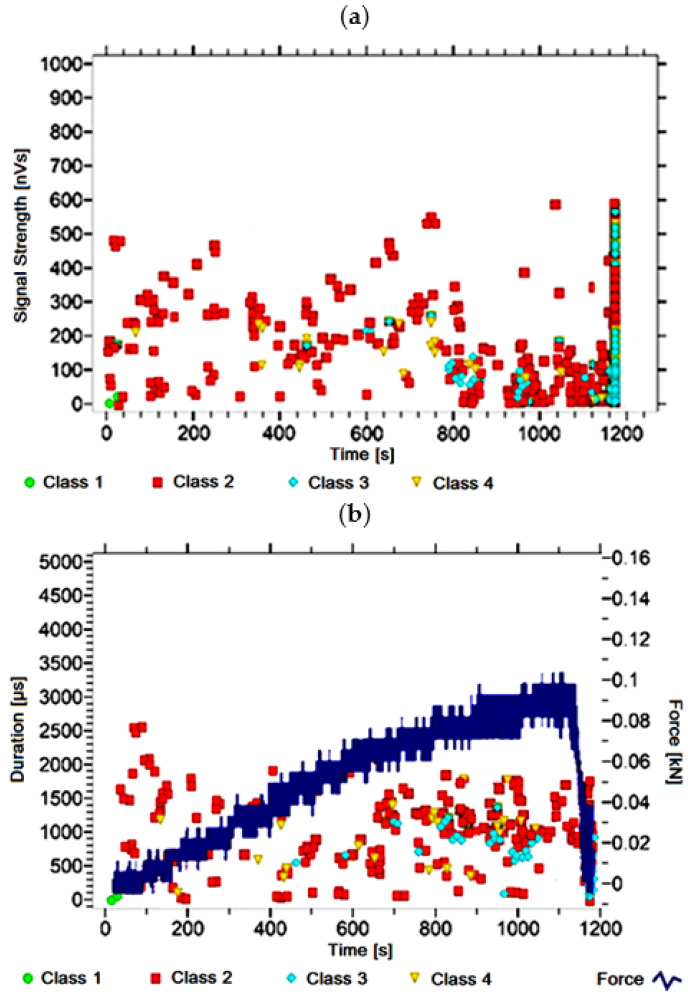
Graphs of acoustic emission signals for an exemplary sample of A_11_ series: (**a**) signal strength over time; (**b**) distribution of the duration of the signals over time with the force increment curve.

For the sample from the A_11_ (Figure 12) test case, signals of 1 and 2 classes were observed for the majority of each of the recorded waveforms. Single class 3 and 4 signals start to appear around 400 s and are emitted when the sample is destroyed. Few signals corresponding to the initiation of microcracks appear from the start of the test until they reach 200 s; their power is up to 170 nVs and their duration up to 250 µs. The signals linked to crack development and spread are recorded throughout the measurement and are characterised by a power of up to 600 nVs and a duration of even up to 2500 µs. Events corresponding to material delamination and fibre detachment are characterised by up to 600 nVs and have a duration of 1500 µs. The power of the signals linked with fibre breakage and component destruction reaches 600 nVs and their duration is 1700 µs.

#### 3.1.12. Signal Analysis for Research Case A_12_

**Figure 13:**
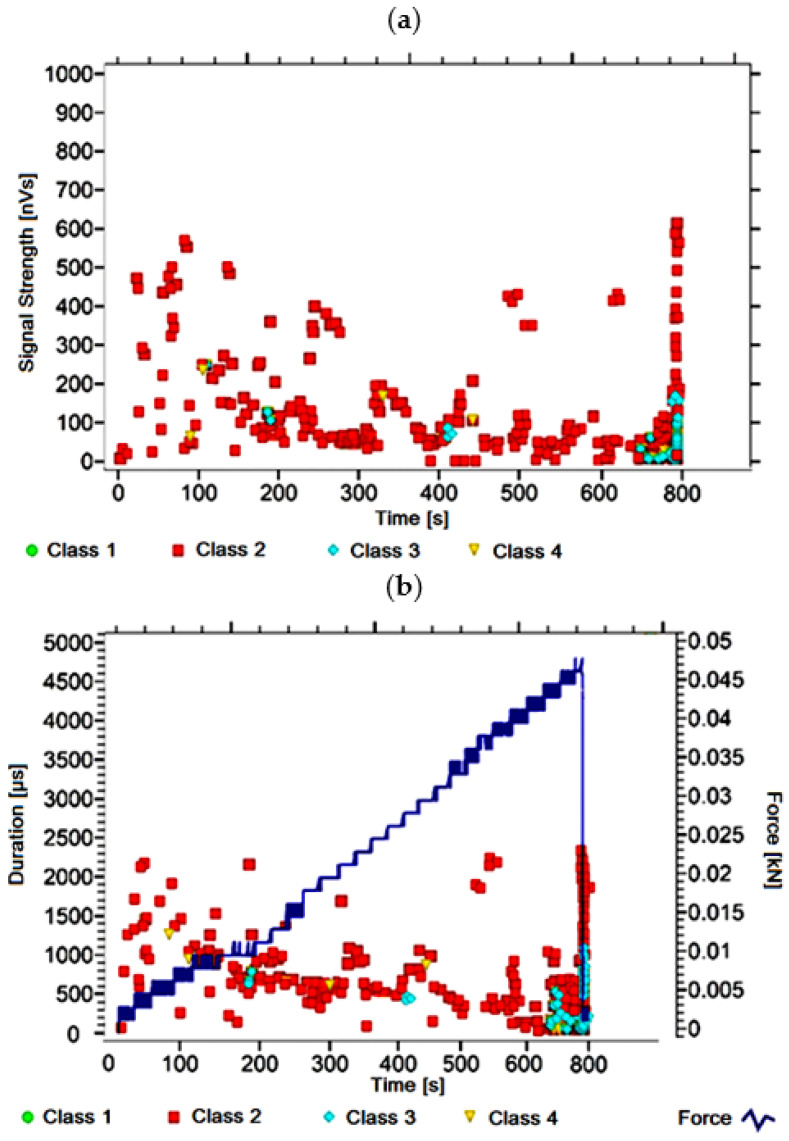
Graphs of acoustic emission signals for an exemplary sample of _A12_ series: (**a**) signal strength over time; (**b**) distribution of the duration of the signals over time with the force increment curve.

For the sample from the A_12_ (Figure 13) test case, signals of 1 and 2 classes were observed for the majority of each of the recorded waveforms. Single 3 and 4 class signals are emitted after 100 s. Few signals corresponding to the initiation of microcracks appear from the start of the test until they reach 200 s; their power is up to 250 nVs and their duration up to 300 µs. The signals linked to crack development and spread are recorded throughout the measurement and are characterised by a power of up to 600 nVs and a duration of even up to 2500 µs. Events corresponding to material delamination and fibre detachment are characterised by up to 250 nVs and have a duration of 1300 µs. The power of the signals linked with fibre breakage and component destruction reaches 250 nVs and their duration is 1300 µs.

#### 3.1.13. Signal Analysis for Research Case A_13_

**Figure 14:**
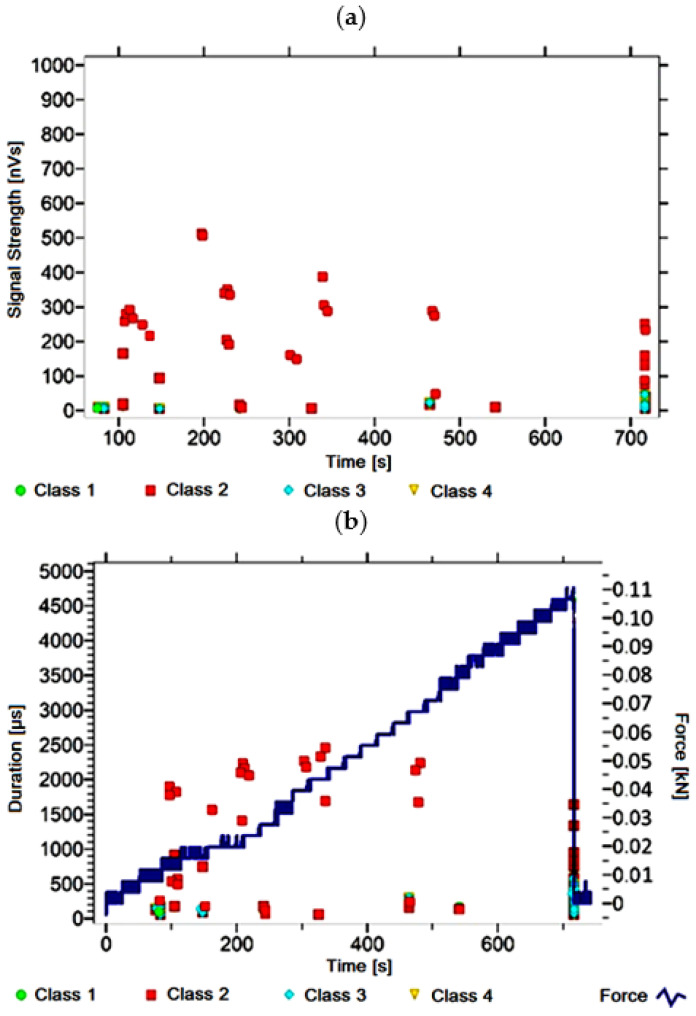
Graphs of acoustic emission signals for an exemplary sample of A_13_ series: (**a**) signal strength over time; (**b**) distribution of the duration of the signals over time with the force increment curve.

For the sample from the A_13_ (Figure 14) test case, signals of class 2 were observed for the majority of the waveform observed. The number of class 1 and 3 signals is small. No class 4 signals were recorded. The few signals corresponding to the initiation of microcracks are characterised by a power of 20 nVs and a duration of up to 120 µs. The signals linked to crack development and spread are recorded throughout the measurement and are characterised by a power of up to 550 nVs and a duration of even up to 2500 µs. Events corresponding to material delamination and fibre detachment are characterised by up to 100 nVs and have a duration of 700 µs.

#### 3.1.14. Signal Analysis for Research Case A_14_

**Figure 15:**
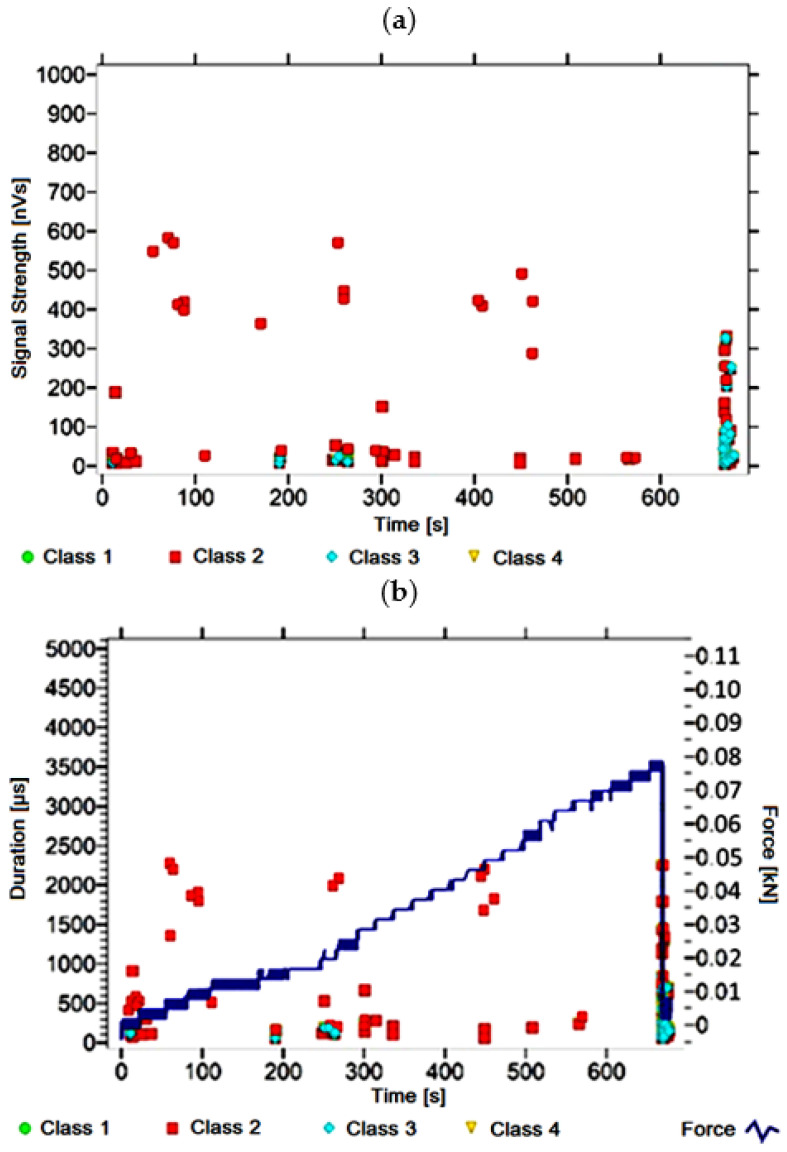
Graphs of acoustic emission signals for an exemplary sample of A_14_ series: (**a**) signal strength over time; (**b**) distribution of the duration of the signals over time with the force increment curve.

For the sample from the A_14_ (Figure 15) test case, signals of class 2 were observed for the majority of the waveform observed. Single class 3 signals begin to appear after 200 s. The presence of class 1 and 4 signals was not recorded. The signals linked to crack development and spread are recorded throughout the measurement and are characterised by a power of up to 600 nVs and a duration of even up to 2500 µs. Events corresponding to material delamination and fibre detachment are characterised by up to 350 nVs and have a duration of up to 1000 µs.

#### 3.1.15. Summary of Signal Analysis

Analysing Figure 2, Figure 3, Figure 4, Figure 5, Figure 6, Figure 7, Figure 8, Figure 9, Figure 10, Figure 11, Figure 12, Figure 13, Figure 14 and Figure 15, it can be seen that for samples in the air-dry state and exposed to environmental factors (A_1_–A_9_) there are signals of all classes from the beginning of the external load action. Shortly after applying the force, class 3 signals begin to appear, indicating that the structural delimination and fibre detachment from the matrix have begun. As the cracks in the tensile zone progresses and the crack deepens, class 4 signals for gradual fibre breakage and material deterioration start to appear at the bottom of the sample. Exceeding the stress limits in the material results in a rapid increase in the recorded descriptors.

For the samples exposed to direct fire (A_10_–A_13_) and high temperature (A_14_), the flame interaction did not affect the nature of the signals recorded for the samples torched for 2.5 min (A_10_), while the prevailing class 2 signals were observed in the other recorded time courses. The author linked this fact to the potential for damage or degradation of the reinforcing fibres when exposed to fire and temperature, which implied a change in the deterioration of the components. It was found that dry samples and samples exposed to environmental conditions, due to the presence of reinforcing fibres, were destroyed by exceeding tensile stresses. On the other hand, the deterioration process of the samples exposed to temperature fluctuations, caused by probable fibre degradation, was caused by exceeding the shear strength. The analysis of the characteristics of the recorded descriptors also revealed that, during the bending of the torched and burned samples, the occurrence of most AE events was associated with the emission of signals with lower power (below 600 nVs) than those of the other groups.

Analysing the classes of AE signals present in the analysed waveforms of A_11_–A_14_ samples, it was found that the absence of class 1 signals in the initial phase of testing indicates the potential presence of locally weakened structure of cement-fibre panels. The absence of the class 3 and 4 signals in the recorded events clearly indicates damage to the reinforcing fibres or the potential delamination and voids in the material structure.

### 3.2. Statistical Analysis of the Obtained Results

During statistical analysis of received results, for all samples, first, all data were pre-tested in order to select the appropriate test groups to examine the data. The groups examined are approximately equinumerous. Therefore, standard distributions of data in individual groups were examined with the Shapiro–Wilk test. For most data, no grounds to reject the hypothesis of standard distribution were found. The Levene test was then performed to examine the homogeneity of variance.

In most groups there is no homogeneous variance. Therefore, in order to examine the distributions, it was decided to use the non-parametric test for Kruskal–Wallis independent variables.

The below charts provide graphical results of the Kruskal–Wallis test.

#### 3.2.1. Kruskal-Wallis Test Results for Independent Samples of the Number of Class 1 Signals

**Figure 16:**
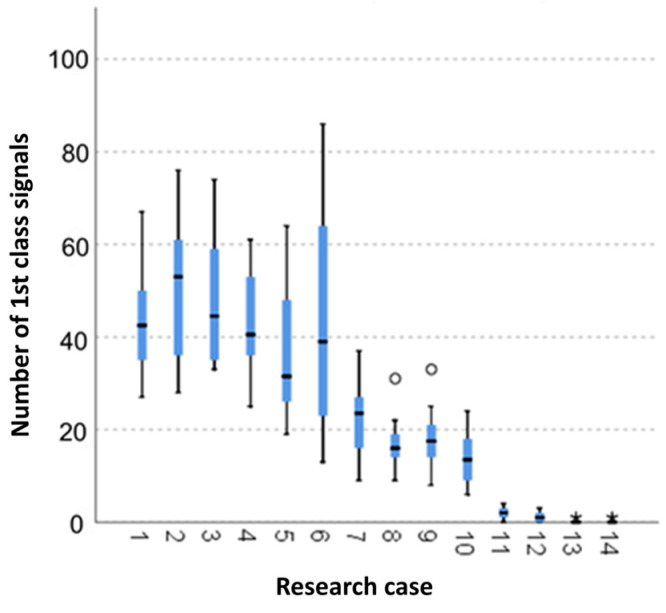
The graphical representation of the Kruskal–Wallis test results for independent samples of the number of class 1 signals.

Analysing the graphical representation of the Kruskal–Wallis test results for independent samples of the number of class 1 signals (Figure 16), it can be observed that the maximum number of signals of this class was recorded for panels from the A_6_ test case (frozen-thawed samples for 10 cycles). For this case there was also the largest scatter of results. The A_8_ test cases (frozen-thawed samples for 50 cycles) and A_9_ (torched samples for 100 cycles) contain single statistical outliers. The lowest values of the class 1 signal number were recorded for the A_13_ test cases (torched samples for 10 min) and A_14_ (burned samples).

#### 3.2.2. Kruskal-Wallis Test Results for Independent Samples of the Average Signal Strength of the Class 1 Signals

**Figure 17:**
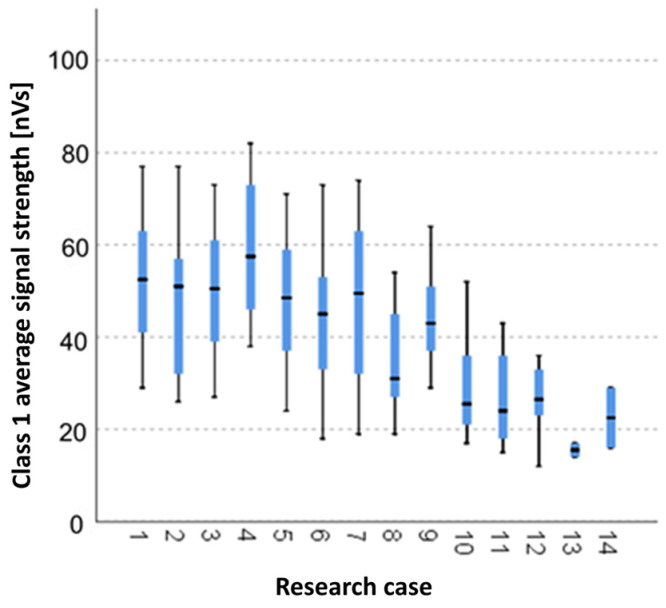
The graphical representation of the Kruskal-Wallis test results for independent samples of the average signal strength of the class 1 signals.

Analysing the graphical representation of the Kruskal–Wallis test results for independent tests of the average signal strength class 1 signals (Figure 17), it can be observed that the maximum mean power of the signals of this class was recorded for panels from the A_4_ test case (soaked-dried samples for 25 cycles). The largest scatter of results occurred for the A_7_ group (frozen-thawed samples for 25 cycles). The lowest value of the average signal strength of class 1 signals was recorded for the A_12_ test case (torched samples for 7.5 min).

#### 3.2.3. Kruskal-Wallis Test Results for Independent Tests of the Average Duration of the Class 1 Signals

**Figure 18:**
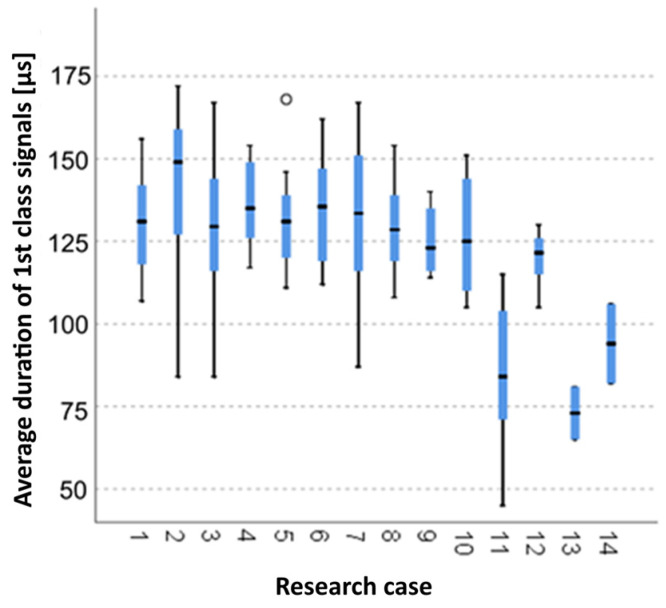
The graphical representation of the Kruskal–Wallis test results for independent tests of the average duration of the class 1 signals.

Analysing the graphical representation of the Kruskal-Wallis test results for independent tests of the average duration of the class 1 signals (Figure 18), it can be observed that the average duration of the signals of this class was recorded for panels from the A_2_ test case (soaked samples for 1 h). Also for this case there was the largest scatter of results. The A_5_ test case (soaked-dried samples for 50 cycles) contains a single statistical outlier. The lowest value of the duration of the class 1 signals was recorded for the A_11_ test case (torched samples for 5 min).

#### 3.2.4. Kruskal-Wallis Test Results for Independent Tests of the Number of Class 2 Signals

**Figure 19:**
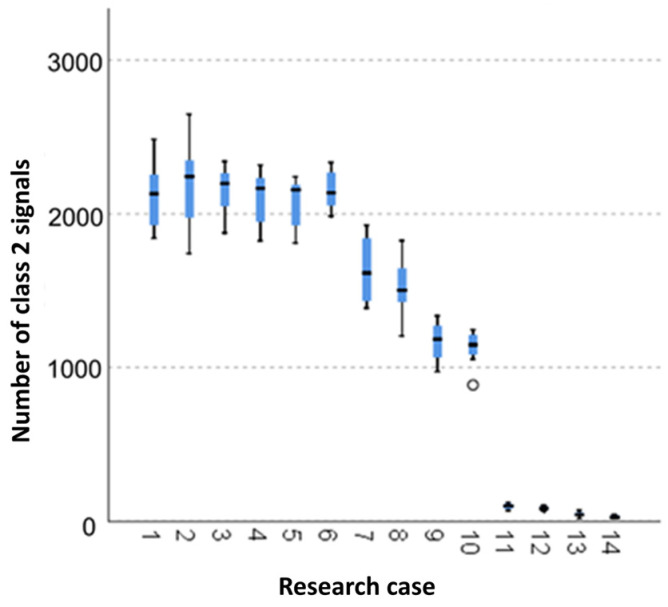
The graphical representation of the Kruskal–Wallis test results for independent tests of the number of class 2 signals.

Analysing the graphical representation of the Kruskal–Wallis test results for independent tests of the number of class 2 signals (Figure 19), it can be observed that the maximum number of signals of this class was recorded for panels from the A_2_ test case (soaked samples for 1 h). Also for this case there was the largest scatter of results. The A_10_ test case (torched samples for 2.5 min) contains a single statistical outlier. The lowest values for the number of class 2 signals were recorded for the A_14_ case (burnt samples).

#### 3.2.5. Kruskal-Wallis Test Results for Independent Samples of the Average Signal Strength of the Class 2 Signals

**Figure 20:**
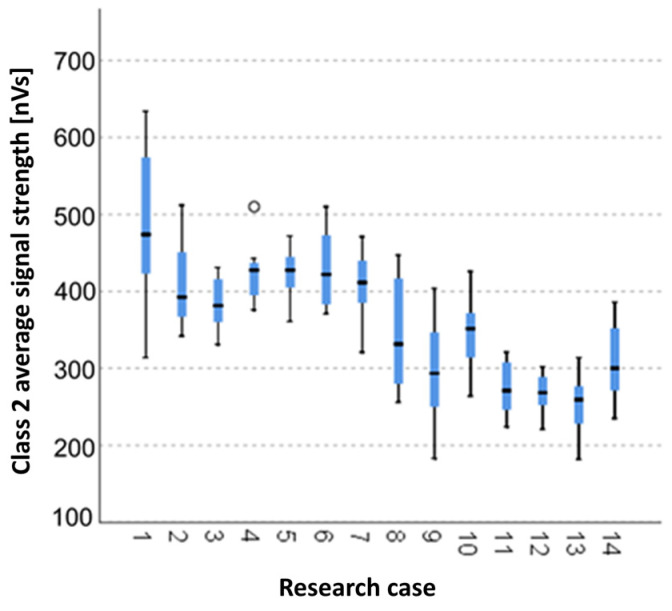
The graphical representation of the Kruskal–Wallis test results for independent samples of the average signal strength of the class 2 signals.

Analysing the graphical representation of the Kruskal–Wallis test results for independent tests of the average signal strength of the class 2 signals (Figure 20), it can be observed that the maximum average signal strength of the signals of this class was recorded for panels from the A_1_ test case (air-dry samples). For this case there was also the largest scatter of results. The A_4_ test case (soaked-dried samples for 25 cycles) contains a single statistical outlier. The lowest value of the average signal strength of the class 2 signals was recorded for the A_13_ test case (torched samples for 10 min).

#### 3.2.6. Kruskal-Wallis Test Results for Independent Tests of the Average Duration of the Class 2 Signals

**Figure 21:**
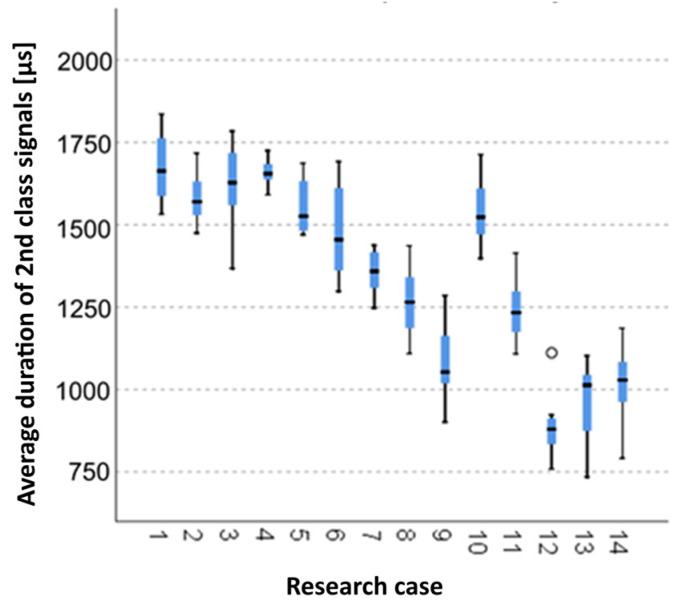
The graphical representation of the Kruskal–Wallis test results for independent tests of the average duration of the class 2 signals.

Analysing the graphical representation of the Kruskal–Wallis test results for independent tests of the average duration of the class 2 signals (Figure 21), it can be observed that the maximum average duration of the signals of this class was recorded for panels from the A_1_ test case (air-dry samples). The largest scatter of results occurred for the A_3_ group (soaked samples for 24 h). The A_12_ test case (torched samples for 10 min) contains a single statistical outlier. The lowest value of the average duration of the class 2 signals was recorded for the A_13_ test case (torched samples for 10 min).

#### 3.2.7. Kruskal-Wallis Test Results for Independent Tests of the Number of the Class 3 Signals

**Figure 22:**
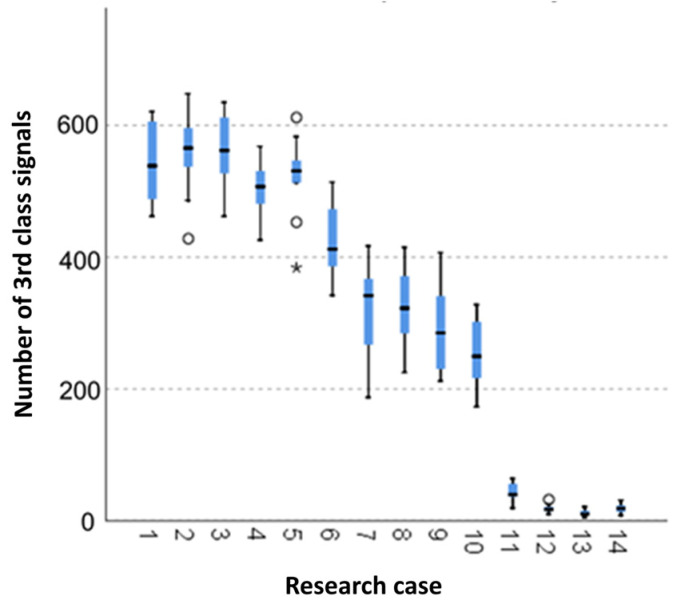
The graphical representation of the Kruskal–Wallis test results for independent tests of the number of the class 3 signals.

Analysing the graphical representation of the Kruskal–Wallis test results for independent tests of the number of class 3 signals (Figure 22), it can be observed that the maximum number of signals of this class was recorded for panels from the A_2_ test case (soaked samples for 1 h). The largest scatter of results occurred for the A_7_ group (frozen-thawed samples for 25 cycles). The A_2_ test case (samples soaked in water for 1 h), A_5_ (samples exposed to soaking-drying for 50 cycles) and A_12_ (torched samples for 7.5 min) contain single statistical outliers. The lowest value of the number of the class 3 signals was recorded for the A_13_ test case (torched samples for 10 min).

#### 3.2.8. Kruskal-Wallis Test Results for Independent Samples of the Average Signal Strength of the Class 3 Signals

**Figure 23:**
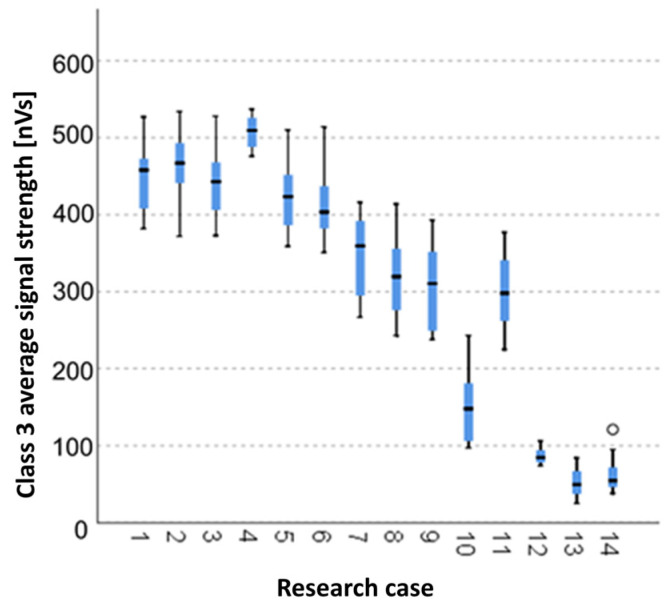
The graphical representation of the Kruskal–Wallis test results for independent samples of the average signal strength of the class 3 signals.

Analysing the graphical representation of the Kruskal–Wallis test results for independent tests of the average signal strength of the class 3 signals (Figure 23), it can be observed that the maximum average signal strength of the signals of this class was recorded for panels from the A_4_ test case (soaked-dried samples for 25 cycles). The largest scatter of results occurred for the A_8_ group (frozen-thawed samples for 50 cycles). The A_14_ test case (burned samples) contains a single statistical outlier. The lowest values of the average signal strength of the class 3 signals was recorded for the A_13_ test case (torched samples for 10 min).

#### 3.2.9. Kruskal-Wallis Test Results for Independent Tests of the Aver-Age Duration of the Class 3 Signals

**Figure 24:**
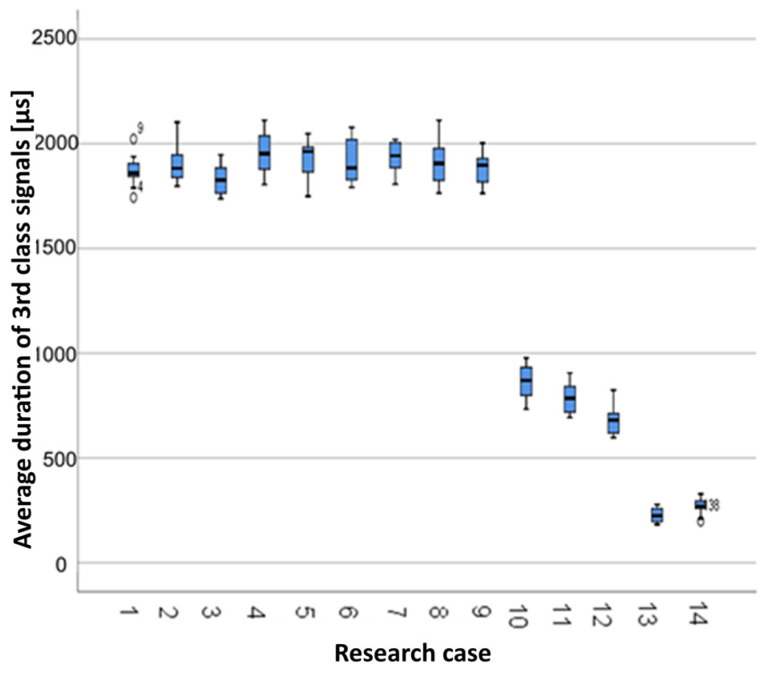
The graphical representation of the Kruskal–Wallis test results for independent tests of the average duration of the class 3 signals.

Analysing the graphical representation of the Kruskal–Wallis test results for independent tests of the average duration of the class 3 signals (Figure 24), it can be observed that the maximum average duration of the signals of this class was recorded for panels from the A_4_ test case (soaked-dried samples for 25 cycles). The largest scatter of results occurred for the A_8_ group (frozen-thawed samples for 50 cycles). The A_1_ test case (air-dry samples) and A_14_ (burned samples) contain single statistical outliers. The lowest value of the average duration of the class 3 signals was recorded for the A_13_ test case (torched samples for 10 min).

#### 3.2.10. Kruskal-Wallis Test Results for Independent Tests of the Number of the Class 4 Signals

**Figure 25:**
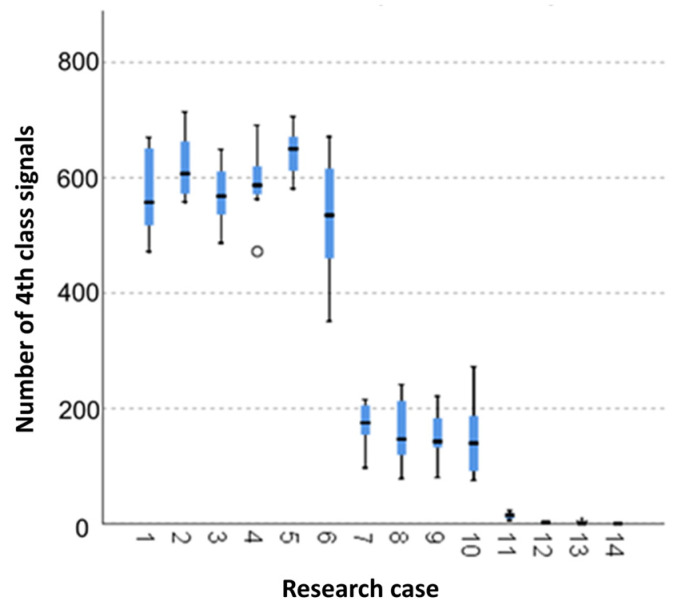
The graphical representation of the Kruskal–Wallis test results for independent tests of the number of the class 4 signals.

Analysing the graphical representation of the Kruskal–Wallis test results for independent tests of the number of class 4 signals (Figure 25), it can be observed that the maximum number of signals of this class was recorded for panels from the A_2_ test case (soaked samples for 1 h). The largest scatter of results occurred for the A_6_ group (frozen-thawed samples for 10 cycles). The A_4_ test case (soaked-dried samples for 50 cycles) contains single statistical outliers. The lowest values for the number of the class 4 signals were recorded for the A_14_ case (burnt samples).

#### 3.2.11. Kruskal-Wallis Test Results for Independent Samples of the Average Signal Strength of the Class 4 Signals

**Figure 26:**
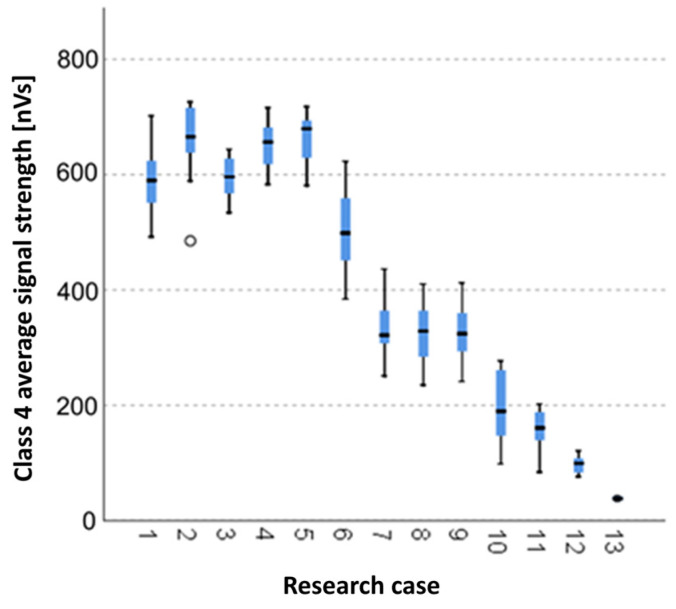
The graphical representation of the Kruskal–Wallis test results for independent samples of the average signal strength of the class 4 signals.

Analysing the graphical representation of the Kruskal–Wallis test results for independent tests of the average signal strength of class 4 signals (Figure 26), it can be observed that the maximum average signal strength of the signals of this class was recorded for panels from the A_2_ test case (soaked samples for 1 h). The largest scatter of results occurred for the A_6_ group (frozen-thawed samples for 10 cycles). The A_2_ test case (soaked samples for 1 h) contains single statistical outliers. The lowest values for the average signal strength of the class 4 signals were recorded for the A_14_ case (burnt samples).

#### 3.2.12. Kruskal-Wallis Test Results for Independent Tests of the Average Duration of the Class 4 Signals

**Figure 27:**
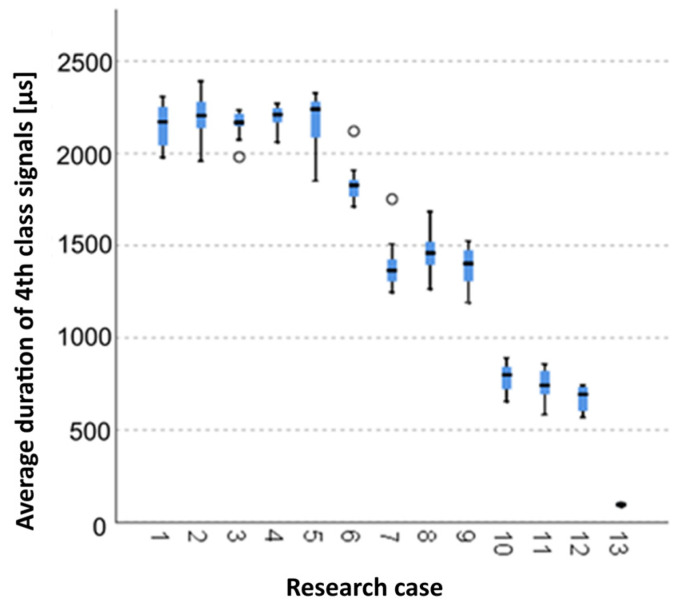
The graphical representation of the Kruskal–Wallis test results for independent tests of the average duration of the class 4 signals.

Analysing the graphical representation of the Kruskal–Wallis test results for independent tests of the average duration class 4 signals (Figure 27), it can be observed that the average duration of the signals of this class was recorded for panels from the A_2_ test case (soaked samples for 1 h). The largest scatter of results occurred for the A_5_ case (soaked-dried samples for 50 cycles). The A_3_ test case (samples soaked in water for 24 h), A_6_ (frozen-thawed samples for 10 cycles) and A_7_ (frozen-thawed samples for 25 cycles) contain single statistical outliers. The lowest values for the average duration of the class 4 signals were recorded for the A_14_ case (burnt samples).

#### 3.2.13. Kruskal-Wallis Test Results for Independent Tests of the *F_max_* Break Force

**Figure 28:**
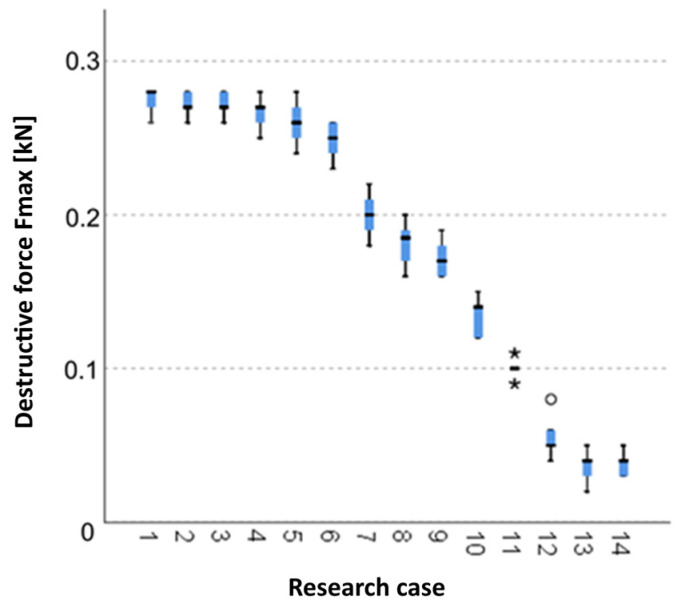
Kruskal–Wallis test results for independent tests of the *F_max_* break force.

Analysing the graphical representation of the Kruskal–Wallis test results for independent tests of the *F_max_* break force (Figure 28), it can be observed that the maximum destructive force was recorded for panels from the A_5_ test case (soaked-dried samples for 50 cycles). For this case there was also the largest scatter of results. The A_11_ test case (burned samples for 50 min.) and A_12_ (torched samples for 7.5 min.) contain single statistical outliers. The lowest value of the *F_max_* break force was recorded for the A_13_ test case (torched samples for 10 min).

#### 3.2.14. Checking the Significance of the Correlation

The Shapiro–Wilk test was conducted to examine the normality of the data distribution. In most cases the data do not have a normal distribution. Therefore, the Spearman correlation coefficient (a non-parametric measure of monotonic statistical dependence between random variables) was used to examine the relationship between the indicated variations.

A significant correlation was found to exist between:

- The number of the class 1 signals and the destructive force *F_max_* (ρ = 0.809, p = 0.000). The more signals, the higher the destructive force.
- The average signal strength of the class 1 signals and the destructive force *F_max_* (ρ = 0.374, p = 0.000). The greater power of the signals, the higher the destructive force.
- The number of class 2 signals and the destructive force *F_max_* (ρ = 0.718, p = 0.000). The more signals, the higher the destructive force.
- The average signal strength of the class 2 signals and the destructive force *F_max_* (ρ = 0.633, p = 0.000). The greater power of the signals, the higher the destructive force.
- The average duration of the class 2 signals and the destructive force *F_max_* (ρ = 0.737, p = 0.000). The greater average duration of the signals, the higher the destructive force.
- The number of the class 3 signals and the destructive force *F_max_* (ρ = 0.667, p = 0.000). The more signals, the higher the destructive force.
- The average signal strength of the class 3 signals and the destructive force *F_max_* (ρ = 0.480, p = 0.000). The greater power of the signals, the higher the destructive force.
- The average duration of the class 3 signals and the destructive force *F_max_* (ρ = 0.493, p = 0.000). The greater average duration of the signals, the greater the destructive force.
- The number of the class 4 signals and the destructive force *F_max_* (ρ = 0.780, p = 0.000). The more signals, the higher the destructive force.
- The average signal strength of the class 4 signals and the destructive force *F_max_* (ρ = 0.735, p = 0.000). The greater power of the signals, the higher the destructive force.
- The average duration of the class 4 signals and the destructive force *F_max_* (ρ = 0.766, p = 0.000). The greater average duration of the signals, the higher the destructive force.

Based on the distribution of the results of Kruskal–Wallis tests for individual parameters and taking into account the significance of the correlation between them, it can be concluded that the differences in the number of recorded acoustic emission signals of particular classes and the parameters of these signals make it possible to create groups characterizing AE events in connection with the mechanical parameters of boards. This is what has been treated as the basis of the methodology for assessing the condition of cement-cellulose boards.

## 4. Discussion

Given the results of the tests and the resulting conclusions indicating the applicability of the acoustic emission method based on signal classification using the *k-means* algorithm for the assessment of variations in the mechanical parameters of cement-fibre composites, a methodology for such assessment was therefore developed. The approach proposed is a reasonable method for assessing the variation in mechanical parameters of fibre-cement panels on the basis of the parameters determined by the non-destructive method indicated. During the development of the methodology, the results of research of the recorded frequencies of events were also used, and an approach to tracking acoustic spectra of signals was included, which, according to the authors, is useful as a refinement of observations.

**Figure 29:**
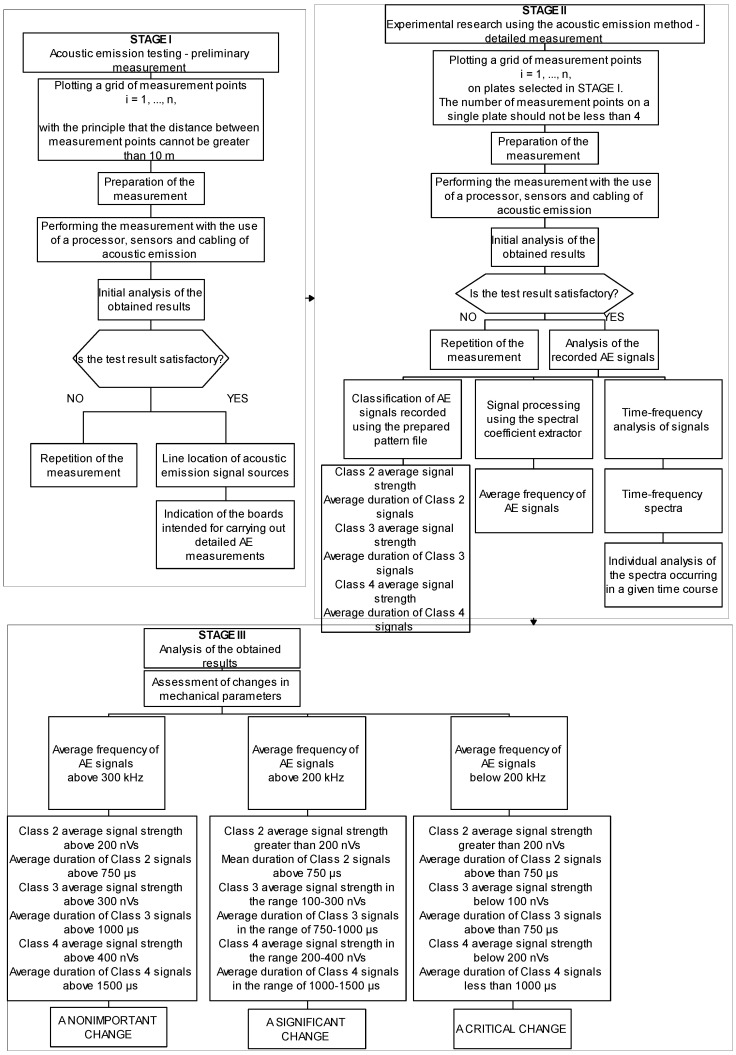
Diagram illustrating the methodology of assessing the change of mechanical parameters of fiber-cement boards under the influence of operational factors.

The suggested methodology is shown schematically on Figure 29. Three stages can be distinguished in this scheme. Stage 1 involves plotting a grid of measurement points on the façade covered with fibre-cement panels at a spacing not exceeding 10 m. The measuring points along the edge of the panel should be located approximately 50 mm from it. The next step is to prepare the acoustic emission measurement, including sensor testing and elimination of background noise. The next step is to take a measurement. Next, it is necessary to perform a linear localization that allows the precise location of the points where AE signals are produced. Based on these steps, it is possible to identify potential panels where mechanical deterioration may have occurred.

Stage 2 involves conducting acoustic emission measurements on the panels selected in stage 1. Taking into account the size of a single fibre-cement panel used as a façade cladding, it is recommended that the number of measurement points on a single panel should be not less than 4. Again, the distance between the AE sensor locations and the edge of the panel must be at least 50 mm. Once the measurement has been prepared, proceed to the recording of the AE signals. Due to the operating nature of the cladding, it is recommended that the signal acquisition is performed at increased strain on the individual components (preferably during relatively strong wind pressure). Once the measurement is complete, the recorded signals must be processed. The first stage of the analysis involves the classification of AE signals using the developed reference file. At this stage of processing, it is important to remember that signals with a coefficient of determination R2 (matching a given class) of at least 90% can be adopted to assess the extent of variation in mechanical parameters. In the next step, the values of the average signal strength and duration of the class 2, 3, and 4 signals have to be analysed.

The final stage (3) is an analysis of the previously obtained results using this testing method with three approaches to process the recorded signals. The analysis is aimed at determining and assessing the extent to which the mechanical parameters change when exposed to the service conditions of the fibre-cement panels tested, based on the results of the conducted tests. Insignificant change in mechanical performance means average signal strength of the class 2 signals over 200 nVs, average duration of the class 2 signals over 750 µs, average signal strength of the class 3 signals over 300 nVs, average duration of the class 3 signals over 150 µs, average signal strength of the class 4 signals over 400 nVs, average duration of the class 4 signals over 1500 µs, and average frequency of AE signals over 300 kHz. Significant change in mechanical performance means average signal strength of the class 2 signals over 200 nVs, average duration of the class 2 signals over 750 µs, average signal strength of the class 3 signals within 100–300 nVs, average duration of the class 3 signals within 100–150 µs, average signal strength of the class 4 signals within 200–400 nVs, average duration of the class 4 signals within 1000–1500 µs, and average frequency of AE signals within 200–300 kHz. Critical change in mechanical performance means average signal strength of the class 2 signals over 200 nVs, average duration of the class 2 signals over 750 µs, average signal strength of the class 3 signals over 100 nVs, average duration of the class 3 signals below 100 µs, average signal strength of the class 4 signals below 200 nVs, average duration of the class 4 signals below 1000 µs, and average frequency of AE signals below 300 kHz.

The insignificant change in mechanical parameters is associated with a reduction in MOR flexural strength of no more than 30% with respect to the reference panels. The significant change is a reduction in strength by more than 30% but less than 50%. Deterioration of strength properties by more than 50% was classified as a critical change in mechanical performance.

The advantages of the presented methodology relate primarily to the general advantages of using the phenomenon of acoustic emission:

- The ability to detect a range of failure mechanisms related to the processes that characterize particular classes of signals in the early stages before they become significant problems;
- The possibility of observation during the operation of the boards;
- The ability to locate the sources of damage and to distinguish them;
- The ability to perform global monitoring of the building object;
- The possibility of assessing the structure in real operating conditions;
- Non-invasive method;
- Possibility of remote monitoring;
- The ability to detect hard-to-reach damage.

The methodology limits are as follows:

- No possibility to detect existing damage that does not develop and propagate;
- Extended monitoring time compared to other non-destructive methods (e.g., ultrasonic method).

## 5. Conclusions

When applying the acoustic emission method, two main approaches are used to analyse the acoustic data: the study of AE signal descriptors and/or waveform analysis. In both approaches, data processing is usually based on the analysis of single descriptors or groups of descriptors. However, given the fact that the process of degradation of a building component or structure is not a deterministic but a random process, such a solution causes problems with identification of physical phenomena causing destructive processes. Therefore, current procedures based on precise load control make it difficult to perform diagnostics on large building components under field conditions. Rather, the methods and procedures currently in use are expected to provide information that allows conclusions to be drawn about the effect of recorded defects on the load-bearing capacity and durability of the panels with cement matrix.

Under the conditions in which cement-fibre cladding panels operate, assessment on the basis of the current criteria based on single parameters of acoustic signals is unlikely to solve the problem, making it necessary to develop a new concept for assessing the extent of destruction of a civil engineering structure.

The methodology presented in this paper is a preliminary version of a reliable assessment of changes in the mechanical parameters of the fibre-cement panels. It is the intention of the authors that, once refined, it will enable panel diagnostics to be performed without the need for any expert training in this area.

## Figures and Tables

**Table 1:** The declared technical parameters of the boards.

**Density**	Dry state	PN-EN 12467	≥1.65	g/cm^3^
**Flexural strength**	Perpendicular	PN-EN 12467	24.0	N/mm^2^
**Flexural strength**	In parallel	PN-EN 12467	18.5	N/mm^2^
**Modulus of elasticity**		PN-EN 12467	12,000	N/mm^2^
**Stretching with humidity**	30–95%		1.0	mm/m
**Porosity**	0–100%		>18	%

## Data Availability

Not applicable.
